# Trustworthiness of randomized trials in endocrinology—A systematic survey

**DOI:** 10.1371/journal.pone.0212360

**Published:** 2019-02-19

**Authors:** José Gerardo González-González, Edgar Gerardo Dorsey-Treviño, Neri Alvarez-Villalobos, Francisco Jesús Barrera-Flores, Alejandro Díaz González-Colmenero, Carolina Quintanilla-Sánchez, Victor M. Montori, Rene Rodriguez-Gutierrez

**Affiliations:** 1 Endocrinology Division, Department of Internal Medicine, University Hospital “Dr. José E. González”, Universidad Autónoma de Nuevo León, Monterrey, México; 2 Plataforma INVEST Medicina UANL—KER Unit Mayo Clinic (KER Unit México), Universidad Autónoma de Nuevo León, Monterrey, México; 3 Research Unit, University Hospital “Dr. José E. González”, Universidad Autónoma de Nuevo León, Monterrey, México; 4 Knowledge and Evaluation Research Unit in Endocrinology, Mayo Clinic, Rochester, MN, United States of America; 5 Division of Endocrinology, Diabetes, Metabolism, and Nutrition, Department of Medicine, Mayo Clinic, Rochester, MN, United States of America; University of Mississippi Medical Center, UNITED STATES

## Abstract

**Background:**

Trustworthy (i.e. low risk of bias) randomized clinical trials (RCTs) play an important role in evidence-based decision making. We aimed to systematically assess the risk of bias of trials published in high-impact endocrinology journals.

**Methods:**

We searched the MEDLINE/PubMed database between 2014 and 2016 for phase 2–4 RCTs evaluating endocrine-related therapies. Reviewers working independently and in duplicate used the Cochrane Risk of Bias Tool (CCRBT) to determine the extent to which the methods reported protected the results of each RCT from bias.

**Results:**

We assessed 292 eligible RCTs, of which 40% (116) were judged to be at low risk, 43% (126) at moderate, and 17% (50) at high risk of bias. Blinding of outcome assessment was the least common domain reported 43% (125), while selective reporting of outcomes was the most common 97% (282). In multivariable analysis, RCTs with a parallel design (OR 2.4; 95% CI; 1.2–4.6) and funded by for-profit sources (OR 2.2; 95% CI; 1.3–3.6) were more likely to be at low risk of bias.

**Conclusions:**

Trustworthy evidence should ultimately shape care to improve the likelihood of desirable patient outcomes. Six out-of 10 RCTs published in top endocrine journals are at moderate/high-risk of bias. Improving this should be a priority in endocrine research.

## Introduction

Well conducted randomized clinical trials (RCTs), should help clinicians, patients, and policymakers make more confident decisions about care. Attention to this so-called core principle of evidence-based medicine (EBM),[1] has supported the critical appraisal of the methods used in RCTs, and has contributed to improve health care.[2–5] Such critical appraisal focuses on recognizing that RCTs often lack sufficient protection against bias that consequently reduces the confidence in their estimates.[6, 7] This confidence creates the trustworthiness for clinicians to apply evidence into patient care.

Trustworthiness in RCTs can be drawn by assuring the transparency of trial’s methods.[7] To do so, several strategies have been adopted to guide researchers in their reporting of methods.[8–11] However, despite these guidance, low quality methodological reporting seems to prevail among several fields of medicine.[12–15] These untrustworthy studies, which reliability is at most questionable, are frequently used by policymakers to develop clinical guidelines, promote an intervention, or generate recommendations often labeled as strong.[16, 17] If patient care should be stemmed from research evidence that mainly draws its recommendations from solid evidence aimed at discover, uncover, or invent treatments that improve their lives, relying in trials in which confidence appear to be obscure by untrustworthy methods opposes the true essence of EBM.[18] This incongruency of developing guidelines and recommendations based on quivery evidence, is prone to over- or underestimate the true effect of an intervention, and may ultimately cause harm to the patient or end up being research waste.[19–21] In this instance, conducting low quality clinical research translates into low quality of evidence that ultimately causes low quality of care for patients.

The extent to which the results of important RCTs of treatments for endocrine conditions are protected against bias and thus are trustworthy, however, remains uncertain. Consequently, we aimed to systematically evaluate the overall risk of bias of endocrine RCTs published in high-impact journals between 2014–2016.

## Material and methods

This systematic review adheres to the Preferred Reporting Items for Systematic Review and Meta-analysis (PRISMA) (**S1 Appendix**).[22]

### Study eligibility criteria

Eligible articles were phase 2 to 4 RCTs enrolling patients with an endocrinopathy (e.g., diabetes, thyroid, obesity, bone metabolism, cardiovascular (lipids)/metabolism, and pituitary-gonadal-adrenal axis) to estimate treatment efficacy, regardless of language of publication or number of participants included in the trial. As our intention was to evaluate potential bias of RCTs, we decided to only include the first report of the trial and exclude all follow-ups or any other observational designs (i.e. extensions from an RCTs) aimed at evaluating RCT population.

### Search strategy

With the help of an experienced librarian (N.A-V.), we comprehensively searched MEDLINE/PubMed using the Cochrane Highly Sensitive Search Strategy[23] for RCTs published between January 2014 to September 2016—the time frame was chosen arbitrarily—in; a) top five impact-factor medical journals (*New England Journal of Medicine*, *The Journal of the American Medical Association [JAMA]*, *Lancet*, *Annals of Internal Medicine*, *and the British Medical Journal [BMJ]*), b) two top diabetes journals (*Diabetologia and Diabetes Care*), c) two top general endocrinology journals *(Lancet Diabetes & Endocrinology and Journal of Clinical Endocrinology and Metabolism [JCEM])*, d) two top cardiovascular journal*s (Journal of the American College of Cardiology and Circulation)*, and e) the five top impact-factor journals of thyroid, pituitary, bone, and obesity journals *(Thyroid*, *Pituitary*, *Journal of Bone and Mineral Research*, *International Journal of Obesity*). All journals were selected based on the 2015 Journal Citation Reports (JCR) [24]. The complete search strategy is provided in the **S2 Appendix**.

### Selection of studies

Two pairs of reviewers working independently and in duplicate reviewed all potentially eligible articles. In order to standardize the reviewers’ judgments based on the aforementioned inclusion and exclusion criteria, a pilot study reviewing 20 articles was performed with discussion until the pairs achieved optimal chance-adjusted inter-reviewer agreement (kappa ≥ 0.8). Disagreements between reviewers were initially resolved by consensus and, when needed, by adjudication by an endocrinologist and methodologist (R.R-G. or V.M.M.).

### Data collection

Using a standardized web-based form (Online Microsoft Excel 2016, Microsoft, Redmont, WA, USA), reviewers working independently and in duplicate used the Cochrane Risk of Bias Tool (CCRBT) to assess the protection against bias afforded by random sequence, allocation concealment, blinding of personnel and participants, blinding of outcome assessment, incomplete outcome data, selective reporting and use of the intention-to-treat analysis.[9] Additionally, we extracted data regarding year of publication, branch of endocrinology, funding, number of centers, type of outcomes (patient-important outcomes or surrogate or laboratory outcomes), analysis of data (intention-to-treat or per protocol), and type of journal, intervention, and design.

### Risk of bias classification

Each of these seven domains was classified as indicative of high, moderate, or low-risk of bias based on specific criteria. For instance, we classified random sequence generation as placing a study at low risk of bias if the method of allocation was explicitly stated in the article (e.g., a computer-based program was used to randomly allocate patients); when the allocation was reported only as random, we classified the level of protection against bias as unclear. RCTs were also considered at low risk of bias when the investigator gathering the data or processing the data (e.g., trial statistician) were reportedly blind to trial allocation (blinded outcome assessor), when outcomes in trials showed no apparent sign of omission or reporting only positive outcomes (selective reporting), when loss to follow-up was <20% (incomplete outcome data) and when analyses adhered to the intention-to-treat principle. A full and detailed description of each domain is provided in the **S1 Table**.

When adequate protection was present across all seven domains or if only one domain was unprotected, we classified the study as at low risk of bias. When >3 domains were classified as having poor or unclear protections against bias, we classified the study as at high risk of bias. All other RCTs were classified as at being at moderate risk of bias.

### Missing data

When data were missing, unclear or incomplete, we searched for this information in the registration record of the trial in clinicaltrials.gov, the Australian New Zealand Clinical Trial Registry (ANZCTR), or the University Hospital Medical Information Network Clinical Trial Registry (UMIN-CTR). If the study was not registered or data was still unavailable, we contacted the corresponding author. After a lapse of 10 days, if no response was received, we excluded the article. Every contact was documented and reported. Additionally, we foresighted that some RCTs would fail to report the study phase. Whenever this happened, we evaluated each RCT and judged whether it had phase 1 properties, and if so, it was excluded, otherwise the study was included and labeled as “Not Reported”.

### Statistical analysis

We used a descriptive analysis to report categorical variables with frequencies and percentages. We used multivariable analysis using a logistic regression model to assess the probability of a study being at low risk of bias (yes/no). Predictors were selected based on previous evidence and included type of intervention (pharmacological vs. nonpharmacological), trial design (parallel vs. other), type of outcome (patient-important outcomes vs. surrogate or laboratory outcomes), funding (non-profit sources vs. for-profit sources), and number of centers (single vs. multicenter).[16, 25, 26] Adjustments were made on these same variables and goodness-of-fit was determined by the c-statistic and Hosmer-Lemeshow test. Additionally, an univariate analysis was performed to assess the impact of adjusted variables. Trials in which data was missing, were excluded from the multivariable analysis. All variables were inputted in a stepwise backward manner and then excluded until a model that best fitted our data was identified. We took a *p* value < .05 as statistically significant; associations were described using odds ratios (ORs) and their associated 95% confidence interval (CI). We used SPSS version 22 (IBM Corp, Armonk, NY, USA) for all statistical analyses.

## Results

**Fig 1:**
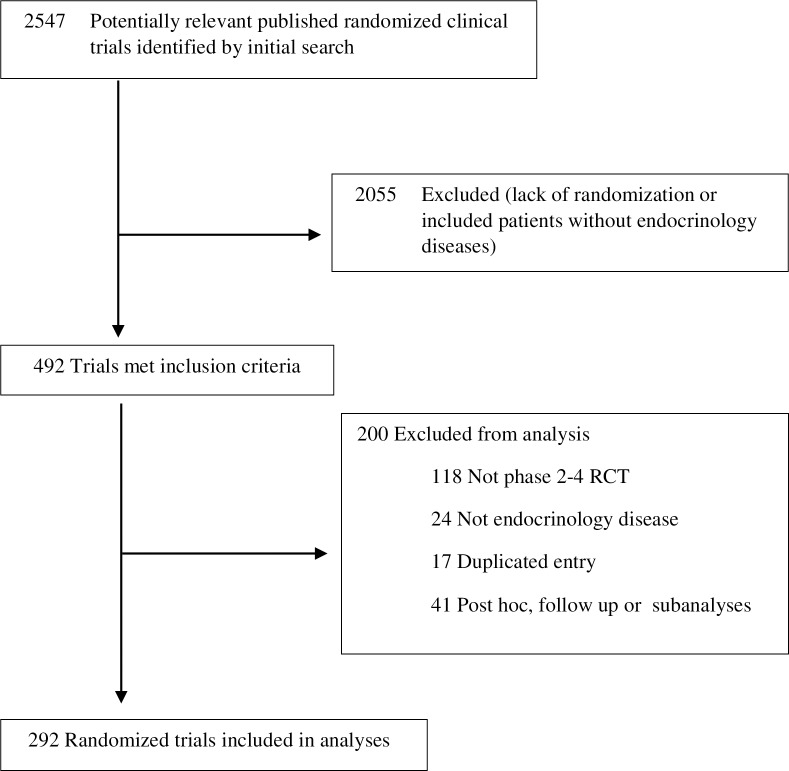
Flow diagram of included studies.

We found 292 eligible RCTs (Fig 1). Most RCTs were industry funded (51%), single-center trials (44%) testing a drug vs. placebo (38%) in patients with diabetes (67%) and measuring its impact on surrogate or laboratory outcomes (70%) (**Table 1**). A complete list of included articles is provided as a supplementary file (**S2 Table**)

**Table 1:** Risk of bias of the 292 included randomized trials.

		* *	Risk of Bias n (%)		
Trial Characteristics		Total	High	Moderate	Low
All Included Articles		292	50 (17)	126 (43)	116 (40)
Year					
	2014	119	23 (19)	45 (37)	51 (42)
	2015	118	18 (15)	57 (48)	43 (36)
	2016	55	9 (16)	24 (43)	22 (40)
Endocrinology Branch					
	Diabetes	195 (67)	33 (17)	81 (41)	81 (41)
	Cardiovascular (Lipids) or Metabolism	42 (14)	3 (7)	22 (52)	17 (40)
	Bone	37 (13)	8 (21)	16 (43)	13 (35)
	Thyroid	10 (3)	2 (20)	6 (60)	2 (20)
	PGA	8 (3)	4 (50)	1 (12)	3 (37)
Type of Intervention					
	Drug vs.				
	Placebo	110 (38)	18 (16)	42 (38)	50 (46)
	Active Drug	76 (26)	14 (18)	36 (47)	26 (34)
	Usual Care	13 (5)	2 (15)	6 (46)	5 (39)
	Other	47 (16)	1 (25)	2 (50)	1 (25)
	Education or Lifestyle Changes	17 (6)	2 (11)	7 (41)	8 (47)
	Nutrition	25 (8.6)	4 (16)	12 (48)	9 (36)
	Other Intervention	47 (16)	9 (19)	21 (44)	17 (36)
Type of Journal					
	Specialty	265 (90)	0 (0)	8 (29.6)	19 (70.4)
	General	27 (9)	50 (18.9)	118 (44.5)	97 (36.6)
Phase					
	2	54 (19)	9 (16)	26 (48)	19 (35)
	3	77 (26)	6 (7)	34 (44)	37 (48)
	4	47 (16)	5 (10)	22 (46)	20 (42)
	Not Reported	114 (39)	30 (26)	44 (38)	40 (35)
Trial Design	Cluster	6	1 (16)	2 (33)	3 (50)
	Crossover	55	20 (16)	26 (47)	9 (16)
	Factorial	3	0 (0)	1 (33)	2 (66)
	Parallel	227	29 (12)	97 (42)	101 (44)
	Other	1	0 (0)	0 (0)	1 (100)
Number of Centers					
	Single Center	129 (44)	36 (28)	53 (41)	40 (31)
	Few (<10 centers)	56 (19)	7 (13)	25 (45)	24 (43)
	Many (>10 centers)	101 (35)	7 (7)	45 (45)	49 (49)
	Not Reported	6 (2)	0 (0)	3 (50)	3 (50)
Type of Outcome					
	Patient-Important Outcomes	89 (31)	7 (7)	40 (44)	42 (47)
	Surrogate or Laboratory Outcomes	203 (70)	43 (21)	86 (42)	74 (36)
Type of Analysis					
	Intention-to-Treat	139 (47)	8 (5)	43 (30)	88 (63)
	Per Protocol	153 (52)	42 (28)	83 (54)	28 (18)
Region of Work					
	Asia	25	1 (4)	17 (68)	7 (28)
	Australia	18	3 (16)	5 (27)	10 (55)
	Europe	69	18 (26)	30 (43)	21 (30)
	North America	78	20 (25)	30 (38)	28 (35)
	South America	3	0	2 (66)	1 (33)
	Multicenter	99	8 (8)	42 (42)	49 (49)
Funding					
	Profit Sources	148 (51)	17 (12)	51 (34)	80 (54)
	Non-Profit Sources	109 (37)	22 (20)	57 (52)	30 (28)
	Mixed Sources	31 (11)	9 (29)	17 (55)	5 (16)
	Not Reported	4 (1)	2 (50)	1 (25)	1 (25)

Data is presented as frequencies (percentages); PGA = pituitary-gonadal-adrenal axis. Low Risk was labeled if seven or six domains were adequately protected. High risk when three or more domain were inadequately protected against bias. All other instances, were judged as Moderate Risk

Chance-adjusted inter-observer agreement for risk of bias judgments ranged from moderate to acceptable (kappa = 0.5 to 0.8). Of the 292 trials, 116 (40%; 95% CI; 34–45) were judged to be at low risk, 126 (43%; 95% CI; 37–48) at moderate, and 50 (17%; 95% CI; 13–21%) at high risk of bias. Blinding of outcomes assessment was the least used protection (43%; 95% CI; 37–49%) and complete reporting the most commonly used protection against bias (97%; 95% CI; 94–99%) (Table 2). In the multivariable analysis, parallel design (OR 2.4; 95% CI; 1.2–4.6) and funded by profitable sources (OR 2.1; 95% CI; 1.2–3.6) were associated with better protection against bias. (Table 3). However, this association was found to be substantially small (R^2^ = 0.1) and the goodness-of-fit test was moderately inadequate (c-statistic = 0.6 and Hosmer and Lemeshow = 0.7).

**Table 2:** Low risk assessment of the seven domains from the Cochrane Risk of Bias Tool.

* *	* *	* *	Low Risk n (%)						
Trials Characteristics		Total	Random Sequence	Allocation Concealment	Blinding of Personnel and Participants	Blinding of Outcome Assessment	Incomplete Outcome Data	Selective Reporting	Intention-to-Treat
All Included Articles		292	214 (73)	189 (65)	248 (85)	125 (43)	225 (77)	282 (97)	139 (48)
Endocrinology Branch									
	Diabetes	195 (67)	142 (66)	124 (65)	168 (67)	70 (56)	159 (70)	189 (67)	108 (77)
	Cardiovascular (Lipids) or Metabolism	42 (14)	33 (15)	32 (16)	37 (14)	24 (19)	31 (13)	41 (14)	14 (10)
	Bone	37 (13)	26 (12)	25 (13)	31 (12)	25 (20)	24 (10)	36 (12)	9 (6)
	Thyroid	10 (3)	9 (4)	4 (2)	7 (2)	3 (2)	6 (2)	10 (3)	3 (2)
	PGA	8 (3)	4 (1)	4 (2)	5 (2)	3 (2)	5 (2)	6 (2)	5 (3)
Type of Intervention									
	Drug vs.								
	Placebo	110 (378)	81 (37)	76 (40)	103 (41)	54 (43)	84 (37)	105 (37)	48 (34)
	Active Drug	76 (26)	53 (24)	45 (23)	59 (23)	24 (19)	59 (26)	74 (26)	42 (30)
	Usual Care	13 (5)	10 (4)	8 (4)	10 (4)	5 (4)	12 (5)	12 (4)	5 (3)
	Other	47 (16)	3 (1)	3 (1)	3 (1)	2 (1)	3 (1)	4 (1)	0 (0)
	Education of Lifestyle Changes	17 (6)	13 (6)	9 (4)	15 (16)	8 (6)	14 (6)	17 (6)	9 (6)
	Nutrition	25 (9)	22 (10)	14 (7)	22 (8)	12 (9)	21 (9)	25 (8)	9 (6)
	Other Intervention	47 (16)	32 (15)	34 (18)	36 (14)	20 (16)	32 (14)	45 (16)	26 (18)
Phase									
	2	54 (19)	35 (16)	33 (17)	46 (18)	21 (16)	45 (20)	54 (19)	30 (21)
	3	77 (26)	67 (31)	60 (31)	70 (28)	32 (25)	59 (26)	76 (27)	39 (28)
	4	47 (16)	37 (17)	36 (19)	39 (15)	22 (17)	35 (15)	46 (16)	22 (15)
	Not Reported	114 (39)	75 (35)	60 (31)	93 (37)	50 (40)	86 (38)	106 (37)	48 (34)
Trial Design									
	Parallel	227 (77)	175 (81)	161 (85)	193 (77)	108 (86)	173 (76)	221 (78)	115 (82)
	Cluster	6 (2)	4 (1)	2 (1)	5 (2)	4 (3)	5 (2)	6 (2)	5 (3)
	Crossover	55 (18)	31 (14)	22 (11)	46 (18)	10 (8)	43 (19)	51 (18)	17 (12)
	Factorial	3 (1)	3 (1)	3 (1)	3 (1)	2 (1)	3 (1)	3 (1)	2 (2)
	Other	1 (0)	1 (0)	1 (0)	1 (0)	1 (0)	1 (0)	1 (0)	0 (0)
Type of Journal									
	General	27 (9)	26 (12)	22 (11)	26 (10)	15 (12)	21 (9)	27 (9)	20 (14)
	Specialty	265 (90)	188 (87)	167 (88)	222 (89)	110 (88)	204 (90)	255 (90)	119 (85)
Number of Centers									
	Single Center	129 (44)	84 (39)	70 (37)	102 (41)	55 (44)	100 (44)	122 (43)	40 (28)
	Few (<10 centers)	56 (19)	42 (19)	36 (19)	51 (20)	20 (16)	43 (19)	56 (19)	37 (26)
	Many (>10 centers)	101 (35)	82 (38)	78 (41)	89 (35)	47 (37)	76 (33)	99 (35)	60 (43)
	Not Reported	6 (2)	6 (2)	5 (2)	6 (2)	3 (2)	6 (2)	5 (1)	2 (1)
Region of Work									
	Asia	25 (8)	23 (10)	19 (10)	16 (6)	13 (10)	21 (9)	24 (8)	9 (6)
	Australia	18 (6)	15 (7)	14 (7)	16 (6)	10 (8)	12 (5)	17 (6)	7 (5)
	Europe	69 (23)	46 (21)	36 (19)	58 (23)	20 (16)	50 (22)	65 (23)	29 (20)
	North America	78 (26)	47 (22)	42 (22)	66 (26)	35 (28)	62 (27)	76 (27)	33 (23)
	South America	3 (1)	3 (1)	3 (1)	3 (1)	1 (1)	3 (1)	3 (1)	0 (0)
	Multicenter	99 (33)	80 (37)	75 (39)	89 (35)	46 (36)	77 (34)	97 (34)	61 (43)
Type of Outcome									
	Patient-Important Outcomes	89 (31)	74 (83)	64 (72)	79 (89)	39 (44)	65 (73)	87 (98)	47 (53)
	Surrogate or Laboratory Outcome	203 (70)	140 (69)	125 (70)	168 (83)	86 (43)	160 (79)	194 (96)	92 (45.3)
Funding									
	Profit Sources	148 (51)	118 (55)	113 (59)	136 (54)	68 (54)	114 (50)	143 (50)	88 (63)
	Non-Profit Sources	109 (39)	76 (35)	62 (32)	87 (35)	48 (38)	85 (37)	106 (37)	37 (26)
	Mixed Sources	31 (11)	17 (7)	11 (5)	24 (9)	7 (5)	24 (10)	29 (10)	14 (10)
	Not reported	4 (1.4)	3 (1)	3 (1)	1 (1)	2 (1)	2 (1)	4 (1)	0 (0)

Data is presented in frequencies (percentages); PGA = pituitary-gonadal-adrenal.

**Table 3:** Predictor variables for trustworthy RCTs.

Predictor Variable	Odds Ratio (95% Confidence Interval) for Low Risk of Bias Trustworthiness	
	Univariate	Multivariable
Design (Other vs. Parallel)	2.67 (1.4–5)	2.40 (1.2–4.6)
Funding (Non-profit vs. Profit)	2.39 (1.4–3.9)	2.16 (1.2–3.6)
Center (Single vs. Multicenter)	1.85 (1.1–3)	1.05 (0.5–1.8)
Type of Intervention (No Drug vs Drug)	1.09 (0.6–1.8)	0.67 (0.3–1.2)
Type of Outcome (SorL vs PIO)	1.55 (0.9–2.5)	1.48 (0.8–2.5)
Phase (<4 vs 4)	1.15 (0.6–2.1)	1.2 (0.6–2.3)
Type of Journal (General vs. Specialty)	0.24 (0.1–0.5)	0.29 (0.1–1.7)

SorL = Surrogate or Laboratory Outcomes; PIO = Patient-Important Outcomes

## Discussion

### Summary of findings

About 4 in 10 endocrine RCTs in top medical journals are adequately protected against bias and thus warrant high confidence in their estimates. Industry-funded parallel design RCTs evaluating drugs exhibited the most methodological protections against bias. Fewer than a third of the reviewed RCTs assessed patient-important outcomes, and almost half of these produced results judged to be at low-risk of bias.

### Strengths and limitations

To our knowledge, this is the most contemporary analysis evaluating the overall trustworthiness of RCTs in endocrinology. Although a newer version of the CCRBT has recently been published, perhaps our results may vary with these new rationales for assessing bias in RCTs.[27] However, because we reviewed only top journals, these results may represent a best-case scenario, assuming that the peer-review process enriches the published record with more trustworthy trials. Conversely, our results may underestimate the protection against bias to the extent that RCT reports fail to report methods that the investigators did implement.[28] Our protocol-driven methods and our reliance on multiple, independent, and reproducible judgments to select trials into the review and to analyze each RCT’s risk of bias using a standardized tool should warrant confidence in our findings.

### Comparison with previous studies

Several fields of medicine have sought to evaluate the quality of reporting methods in a variety of fields of medicine.[29–32] Regarding endocrinology, it appears that it has passed more than 20 years and the reporting of methods appears to have barely improve. For instance, in 1996, McIver et. al. assessed the quality of methods in 79 trials enrolling patients with type 2 diabetes and found that the methods used for randomization and blinding were reported in only 15% and 46%, respectively.[33] Ten years later, Montori et al. evaluated 199 diabetes trials and judged 53% of these to be at high risk of bias.[26] Although they did not use the CCRBT, that study also found trials funded by industry to exhibit more protection against bias.[26] More recently, in 2008, Rios et al. broaden the scope and analyzed 89 trials published in three top endocrinology journals (e.g. Journal of Clinical Endocrinology and Metabolism [JCEM], European Journal of Endocrinology, and Clinical Endocrinology) and found that the reporting of allocation concealment (10%), blinding (20%), and intention-to-treat analysis (16%) was suboptimal.[34] Although we found an improvement in the reporting of methods, when analyzing these domains as a whole to determine the overall trustworthiness rendered by trials, we found that endocrine trials warrant untrustworthy results due to insufficient protection against bias. This lack of confidence in endocrine trials resonates with what stated by Brito et. al. in their systematic review, showing that most clinical guidelines recommendations in endocrinology were supported by evidence warranting low confidence.[35]

### Implications for clinical practice and research

For healthcare, to assure that patients receive optimal care that ultimately seeks to improve their lives, that care must be supported by trustworthy evidence. Clinical recommendations are a way in which the healthcare enterprise assures that clinicians can make confident decisions regarding patients’ care.[36, 37] Thus, this so called evidence-based practice should be based on steadfast evidence that warrants confidence and aims at improving patients’ needs and preferences. Nonetheless, it appears, based on our results, that RCTs are predominantly being conducted without any proper protection against bias, which consequently causes clinical guidelines to draw recommendations based on estimates warranting low confidence and directed at improving intangible surrogate markers that render little or no benefit for patients.[38, 39] These incongruencies in clinical research and practice seems to obviate the main purpose of evidence-based medicine, assuring patient’s wellbeing. In light of this situation, clinicians should be more judicious in the confidence inputted in studies or recommendations they use to provide care.

Furthermore, our findings demonstrate that most investigator initiated RCTs, particularly those funded by federal agencies and foundations are associated with features that place their results at high risk of bias. There is evidence that industry funded trials, although more protected from bias, they are more likely to be affected by spin features—the distortion introduced by subtle features related to the trial question (e.g., selection of patients, interventions, outcomes, and methods) and to the presentation of its results—that mislead readers and are harder to ascertain.[40] Some of these concerns are being addressed by a series of efforts that include initiatives to promote better standards,[41] prospectively register trials,[42, 43] publish all the results from all trials,[44] and report trial results in adherence to the Consolidated Standards of Reporting Trials (CONSORT) reporting statement.[45, 46]. The extent to which these initiatives are improving the evidence base for endocrine practice appears limited at this point, and over the last decade. Even efforts to determine the questions that require better evidence derived from systematic guideline development appear futile: a systematic survey found that only 25% of the research gaps identified by The Endocrine Society guideline recommendations as based on evidence warranting low or very low confidence were being tested in ongoing RCTs.[47]

## Conclusion

Most of the RCTs in endocrinology published in top medical journals seem insufficiently protected against bias. Improving the methodological quality of RCTs should be a top priority in endocrine research.

## Supporting information

S1 Appendix PRISMA checklist. (DOC)

S2 Appendix Full search strategy. (DOCX)

S1 Table Detailed description of the Cochrane Risk of Bias Tool domains. (DOCX)

S2 Table List of included articles. (DOCX)
