# Supplementary material for: Trustworthiness of randomized trials in endocrinology—A systematic survey
Source: PLoS One. 2019 Feb 19;14(2):e0212360. doi: 10.1371/journal.pone.0212360 (PMC6380622; doi:10.1371/journal.pone.0212360)
Supplement: S2 Appendix — (DOCX) [file pone.0212360.s002.docx]

**Full Search Startegy**

("Lancet"[Journal] OR "N Engl J Med"[Journal] OR "jama"[Journal] OR "BMJ"[Journal] OR "diabetes care"[Journal] OR "diabetologia"[Journal] OR "circulation"[Journal] OR "Thyroid"[Journal] OR "Annals of internal medicine"[Journal] OR "lancet diabetes endocrinol"[Journal] OR "pituitary"[Journal] OR "International journal of obesity"[Journal] OR "J Clin Endocrinol Metab"[Journal]) AND (((randomized controlled trial[pt] OR controlled clinical trial[pt] OR "randomized controlled trials"[All Fields] OR "random allocation"[All Fields] OR "double blind method"[All Fields] OR "single blind method"[All Fields]) NOT ("animals"[MeSH Terms] NOT ("humans"[MeSH Terms] AND "animals"[MeSH Terms]))) OR (clinical trial[pt] OR "clinical trials as topic"[MeSH Terms]) OR clin[tw] OR clin's[tw] OR clin28[tw] OR clin42[tw] OR clin63[tw] OR clina[tw] OR clinac[tw] OR clinac2100ix[tw] OR clinac21ex[tw] OR clinac2300[tw] OR clinacanthans[tw] OR clinacanthus[tw] OR clinacix[tw] OR clinacoside[tw] OR clinacox[tw] OR clinacs[tw] OR clinacteric[tw] OR clinad[tw] OR clinadamycin[tw] OR clinadapt[tw] OR clinadil[tw] OR clinadmaycin[tw] OR clinadmycin[tw] OR clinafarm[tw] OR clinafloxacin[tw] OR clinafloxacine[tw] OR clinafloxocin[tw] OR clinafoxacin[tw] OR clinagel[tw] OR clinahl[tw] OR clinaical[tw] OR clinal[tw] OR clinalert[tw] OR clinalfa[tw] OR clinalities[tw] OR clinality[tw] OR clinally[tw] OR clinals[tw] OR clinaltrials[tw] OR clinaly[tw] OR clinalyzer[tw] OR clinalyzers[tw] OR clinamen[tw] OR clinamides[tw] OR clinandrium[tw] OR clinantheae[tw] OR clinanthus[tw] OR clinar[tw] OR clinard[tw] OR clinarray[tw] OR clinarrays[tw] OR clinarthrosis[tw] OR clinas[tw] OR clinatec[tw] OR clination[tw] OR clinazolam[tw] OR clinb[tw] OR clinbelg[tw] OR clinbiochem[tw] OR clinbritic[tw] OR clinc[tw] OR clinca[tw] OR clincal[tw] OR clincalfeatures[tw] OR clincally[tw] OR clincalstudyresult[tw] OR clincaltrails[tw] OR clincaltrals[tw] OR clincaltrial[tw] OR clincaltrials[tw] OR clincamycine[tw] OR clincapture[tw] OR clincare[tw] OR clinccal[tw] OR clinch[tw] OR clinch'[tw] OR clinch's[tw] OR clinche[tw] OR clincheck[tw] OR clincheck'[tw] OR clinchecks[tw] OR clinched[tw] OR clinchek[tw] OR clinchem[tw] OR clincher[tw] OR clinchers[tw] OR clinches[tw] OR clinches'[tw] OR clinching[tw] OR clinchy[tw] OR clinci[tw] OR clincial[tw] OR clincially[tw] OR clincialtrails[tw] OR clincialtrials[tw] OR clincian[tw] OR clincians[tw] OR clincians'[tw] OR clincic[tw] OR clincical[tw] OR clincicaltrials[tw] OR clincila[tw] OR clincio[tw] OR clincis[tw] OR clincke[tw] OR clinckemalie[tw] OR clincker[tw] OR clinckers[tw] OR clincl[tw] OR clinclowns[tw] OR clinclowns'[tw] OR clincn[tw] OR clinco[tw] OR clincobiologic[tw] OR clincocytological[tw] OR clincom[tw] OR clincopatholigic[tw] OR clincopathologic[tw] OR clincopathological[tw] OR clincopathology[tw] OR clincopharmacologic[tw] OR clincophathologic[tw] OR clincoradiologic[tw] OR clincoradiological[tw] OR clincostatistical[tw] OR clincotherapeutic[tw] OR clincr[tw] OR clincrnas[tw] OR clincs[tw] OR clinct[tw] OR clincyto[tw] OR clind[tw] OR clinda[tw] OR clindacin[tw] OR clindactyly[tw] OR clindagel[tw] OR cli%[All Fields] AND ((Clinical Trial[ptyp] OR Clinical Trial, Phase III[ptyp] OR Clinical Trial, Phase IV[ptyp] OR Controlled Clinical Trial[ptyp] OR Randomized Controlled Trial[ptyp]) AND ("2014/01/01"[PDAT] : "2016/12/31"[PDAT]) AND "humans"[MeSH Terms]))
