# Supplementary material for: Trustworthiness of randomized trials in endocrinology—A systematic survey
Source: PLoS One. 2019 Feb 19;14(2):e0212360. doi: 10.1371/journal.pone.0212360 (PMC6380622; doi:10.1371/journal.pone.0212360)
Supplement: S1 Table — (DOCX) [file pone.0212360.s003.docx]

| **Table 1.** Detailed description of the Cochrane Risk of Bias Tool Domains. | | | | |
| --- | --- | --- | --- | --- |
| **Domain** | **Description** | **Examples** | | |
|  |  | **Low Risk** | **High Risk** | **Unclear** |
| Random Sequence Generation | Described the method used to generate the allocation sequence with enough detail to allow a proper assesment | Using a computer program | Randomization was based on the day of the week | Not enough detail or description to reach proper judgement |
|  |  | Coin tossing | Based on even or odd days |  |
|  |  | Throwing dice |  |  |
| Allocation Concealment | Describe with enough detail the method used for concealement in order to properly judge if allocation coudl have been forseen | Allocation of a person not involved in the study | A list was provided for researchers to know the allocation | Not enough detail or description to reach proper judgement |
|  |  | Using an external company | Personnel knew the sequence of allocation before enrollment |  |
|  |  | Envelopes with non-transluminal material |  |  |
| Blinding of Participants and Personnel | Description with a clear statement declaration of any method used for blinding the participants and personnel from any knowledge regarding the intervention | Participants and personnel involved were blinded to treatment allocation | Open-label studies | Not enough detail or description to reach proper judgement |
| Blinding of Outcome Assesment | A clear statement mentioning the blinding of the person assessing the outcome. | Assesment of our primary and secondary outcomes were evaluated by a third person blinded to treatment allocation | Evaluations of outcomes were performed by the research team | Not enough detail or description to reach proper judgement |
| Incomplete Outcome Data | Based on the population lost during the duration of the trial | A loss greater than 20% | A loss less than 20% | Not enough detail or description to reach proper judgement |
| Selective Reporting | A clear reporting of all specified outcomes | All outcomes are fully described | Signs of omission of outcomes or spin is evident in the manuscript | Not enough detail or description to reach proper judgement |
