# Supplementary material for: Trustworthiness of randomized trials in endocrinology—A systematic survey
Source: PLoS One. 2019 Feb 19;14(2):e0212360. doi: 10.1371/journal.pone.0212360 (PMC6380622; doi:10.1371/journal.pone.0212360)
Supplement: S2 Table — (DOCX) [file pone.0212360.s004.docx]

| **Table 2.** List of Included Articles. |
| --- |
| 10.1002/jbmr.2351 |
| 10.1002/jbmr.2464] |
| 10.1002/jbmr.2074 |
| 10.1002/jbmr.2390 |
| 10.1002/jbmr.2292 |
| 10.1002/jbmr.2799 |
| 10.1002/jbmr.2701 |
| 10.1002/jbmr.2688 |
| 10.1002/jbmr.2014 |
| 10.1002/jbmr.2822 |
| 10.1002/jbmr.2015 |
| 10.1002/jbmr.2255 |
| 10.1002/jbmr.2448 |
| 10.1002/jbmr.2292 |
| 10.1002/jbmr.2554 |
| 10.1002/jbmr.2442 |
| 10.1002/jbmr.2080 |
| 10.1002/jbmr.2499 |
| 10.1002/jbmr.2157 |
| 10.1002/jbmr.2111 |
| 10.1002/jbmr.2102 |
| 10.1002/jbmr.2010 |
| 10.1161/CIRCULATIONAHA.115.016857 |
| 10.1161/CIRCULATIONAHA.114.011732 |
| 10.1016/j.jacc.2015.10.033 |
| 10.1016/j.jacc.2014.03.019 |
| 10.1016/j.jacc.2014.03.018 |
| 10.1016/j.jacc.2013.04.105 |
| <http://dx.doi.org/10.1016/j.jacc.2014.08.045> |
| <http://dx.doi.org/10.1016/j.jacc.2016.02.014> |
| 10.1016/j.jacc.2016.03.528 |
| <http://dx.doi.org/10.1016/j.jacc.2015.05.065> |
| <http://dx.doi.org/10.1016/j.jacc.2015.08.014> |
| 10.2337/dc13-2411 |
| 10.2337/dc15-0165 |
| 10.2337/dc15-2298 |
| 10.2337/dc13-1245 |
| 10.2337/dc15-0332 |
| 10.2337/dc13-0816 |
| 10.2337/dc13-2159 |
| 10.2337/dc13-2291 |
| 10.2337/dc15-0811 |
| 10.2337/dc15-0779 |
| 10.2337/dc15-1643 |
| 10.2337/dc14-0845 |
| 10.2337/dc15-1136 |
| 10.2337/dc14-0001 |
| 10.2337/dc14-2830 |
| 10.2337/dc15-0781 |
| 10.2337/dc14-1474 |
| 10.2337/dc14-1412 |
| 10.2337/dc13-2664 |
| 10.2337/dc14-1431 |
| 10.2337/dc13-2480 |
| 10.2337/dc14-1585 |
| 10.2337/dc14-1599 |
| 10.2337/dc14-2364 |
| 10.2337/dc13-2161 |
| 10.2337/dc15-0160 |
| 10.2337/dc13-2908 |
| 10.2337/dc13-0842 |
| 10.2337/dc14-1984 |
| 10.2337/dc14-0785 |
| 10.2337/dc14-0666 |
| 10.2337/dc14-0315 |
| 10.2337/dc13-0467 |
| 10.2337/dc13-2911 |
| 10.2337/dc15-2078 |
| 10.2337/dc14-2689 |
| 10.2337/dc13-1636 |
| 10.2337/dc14-1142 |
| 10.2337/dc14-0761 |
| 10.2337/dc13-1544 |
| 10.2337/dc14-0398 |
| 10.2337/dc13-2990 |
| 10.2337/dc14-2629 |
| 10.2337/dc13-2977 |
| 10.2337/dc13-2845 |
| 10.2337/dc15-1274 |
| 10.2337/dc15-0323 |
| 10.2337/dc15-1899 |
| 10.2337/dc14-3101 |
| 10.2337/dc14-2548 |
| 10.2337/dc14-2206 |
| 10.2337/dc15-1730 |
| 10.2337/dc14-1237 |
| 10.2337/dc13-2760 |
| 10.2337/dc13-2759 |
| 10.2337/dc13-2761 |
| 10.2337/dc14-1044 |
| 10.2337/dc14-1625 |
| 10.2337/dc14-1038 |
| 10.2337/dc14-2868 |
| 10.2337/dc13-2855 |
| 10.2337/dc13-2105 |
| 10.2337/dc14-1096 |
| 10.2337/dc13-1672 |
| 10.2337/dc13-1473 |
| 10.2337/dc13-0690 |
| 10.2337/dc13-2955 |
| 10.2337/dc15-2189 |
| 10.2337/dc15-0761 |
| <https://doi.org/10.2337/dc13-1030> |
| 10.2337/dc13-1813 |
| 10.2337/dc14-0876 |
| 10.2337/dc15-2782 |
| 10.2337/dc13-3007 |
| 10.2337/dc14-0890 |
| 10.2337/dc14-0024 |
| 10.2337/dc15-2001 |
| 10.2337/dc14-0327 |
| 10.2337/dc13-3055 |
| 10.2337/dc14-2852 |
| 10.2337/dc13-2918 |
| 10.2337/dc14-2472 |
| 10.2337/dc15-0075 |
| 10.2337/dc14-2365 |
| 10.2337/dc15-1736 |
| 10.2337/dc12-2704 |
| 10.2337/dc13-1771 |
| 10.2337/dc15-1498 |
| 10.2337/dc14-1958 |
| 10.2337/dc15-0772 |
| 10.2337/dc14-0893 |
| 10.2337/dc15-2068 |
| 10.2337/dc14-0835 |
| 10.2337/dc15-0249 |
| 10.2337/dc14-0991 |
| 10.2337/dc14-0990 |
| 10.2337/dc15-1057 |
| 10.2337/dc15-0801 |
| 10.2337/dc16-0014 |
| 10.2337/dc14-3053 |
| 10.2337/dc13-2956 |
| 10.2337/dc15-1531 |
| 10.2337/dc15-0572 |
| 10.2337/dc14-1796 |
| 10.2337/dc15-2344 |
| 10.2337/dc15-2815 |
| 10.2337/dc14-0596 |
| 10.2337/dc14-0030 |
| 10.2337/dc15-0360 |
| 10.2337/dc15-1025 |
| 10.2337/dc15-2145 |
| 10.2337/dc14-1514 |
| 10.2337/dc14-2806 |
| 10.2337/dc14-0684 |
| 10.2337/dc15-2137 |
| 10.2337/dc15-1254 |
| 10.2337/dc14-1416 |
| 10.2337/dc13-2349 |
| 10.2337/dc15-1037 |
| 10.2337/dc15-0171 |
| 10.2337/dc13-2900 |
| 10.2337/dc14-0930 |
| 10.1007/s00125-014-3168-1 |
| 10.1007/s00125-016-3938-z |
| 10.1007/s00125-015-3689-2 |
| 10.1007/s00125-015-3655-z |
| 10.1007/s00125-014-3253-5 |
| 10.1007/s00125-014-3293-x |
| 10.1007/s00125-014-3313-x |
| 10.1007/s00125-015-3648-y |
| 10.1007/s00125-015-3754-x |
| 10.1007/s00125-015-3733-2 |
| 10.1007/s00125-015-3713-6 |
| 10.1007/s00125-016-3903-x |
| 10.1007/s00125-015-3618-4 |
| 10.1007/s00125-016-3874-y |
| 10.1007/s00125-014-3360-3 |
| 10.1007/s00125-015-3741-2 |
| 10.1007/s00125-015-3524-9 |
| 10.1007/s00125-014-3305-x |
| 10.1007/s00125-016-3981-9 |
| 10.1007/s00125-014-3457-8 |
| 10.1007/s00125-014-3334-5 |
| 10.1007/s00125-015-3854-7 |
| 10.1007/s00125-014-3236-6 |
| 10.1007/s00125-014-3483-6 |
| 10.1007/s00125-015-3592-x |
| 10.1007/s00125-013-3127-2 |
| 10.1007/s00125-014-3399-1 |
| 10.1007/s00125-013-3149-9 |
| 10.1210/jc.2013-3919 |
| 10.1210/jc.2015-3279 |
| 10.1210/jc.2014-1371 |
| 10.1210/jc.2015-1443 |
| 10.1210/jc.2015-1520 |
| 10.1210/jc.2015-4146 |
| 10.1210/jc.2014-4348 |
| 10.1210/jc.2013-4151 |
| 10.1210/jc.2015-4097 |
| 10.1210/jc.2015-1541 |
| 10.1210/jc.2015-1715 |
| 10.1210/jc.2016-1801 |
| 10.1210/jc.2015-4181 |
| 10.1210/jc.2015-4062 |
| 10.1210/jc.2015-2335 |
| 10.1210/jc.2015-3952 |
| 10.1210/jc.2015-2839 |
| 10.1210/jc.2015-3698 |
| 10.1210/jc.2015-1643 |
| 10.1210/jc.2014-3718 |
| 10.1210/jc.2015-2803 |
| 10.1210/jc.2013-3665 |
| 10.1210/jc.2014-3014 |
| 10.1210/jc.2015-1860 |
| 10.1210/jc.2015-1176 |
| 10.1210/jc.2015-4063 |
| 10.1210/jc.2014-1101 |
| 10.1210/jc.2015-2352 |
| 10.1210/jc.2015-3591 |
| 10.1210/jc.2015-3906 |
| 10.1210/jc.2014-2186 |
| 10.1210/jc.2016-2530 |
| 10.1210/jc.2014-4113 |
| 10.1210/jc.2014-3802 |
| 10.1210/JC.2015-1542 |
| 10.1210/jc.2015-3003 |
| 10.1210/jc.2015-1531 |
| 10.1210/jc.2014-1837 |
| 10.1210/jc.2014-3792 |
| 10.1210/jc.2015-1996 |
| 10.1210/jc.2014-4244 |
| 10.1210/jc.2015-1026 |
| 10.1210/jc.2015-3084 |
| 10.1210/jc.2015-2070 |
| 10.1038/ijo.2014.177 |
| 10.1210/jc.2015-3415 |
| 10.1210/jc.2015-4013 |
| 10.1210/jc.2015-2580 |
| 10.1210/jc.2016-1631 |
| 10.1210/jc.2016-2217 |
| <http://dx.doi.org/10.1016/S2213-8587(15)00335-6> |
| <http://dx.doi.org/10.1016/S2213-8587(13)70021-4> |
| <http://dx.doi.org/10.1016/S2213-8587(15)00417-9> |
| <http://dx.doi.org/10.1016/S2213-8587(14)70226-8> |
| <http://dx.doi.org/10.1016/S2213-8587(14)70120-2> |
| <http://dx.doi.org/10.1016/S2213-8587(15)00041-8> |
| <http://dx.doi.org/10.1016/S2213-8587(15)00489-1> |
| <http://dx.doi.org/10.1016/S2213-8587(13)70155-4> |
| <http://dx.doi.org/10.1016/S2213-8587(15)00219-3> |
| <http://dx.doi.org/10.1016/S2213-8587(13)70204-3> |
| <http://dx.doi.org/10.1016/S2213-8587(14)70073-7> |
| <http://dx.doi.org/10.1016/S2213-8587(14)70030-0> |
| <http://dx.doi.org/10.1016/S2213-8587(14)70174-3> |
| <http://dx.doi.org/10.1016/S2213-8587(13)70208-0> |
| <http://dx.doi.org/10.1016/S2213-8587(14)70006-3> |
| <http://dx.doi.org/10.1016/S2213-8587(14)70114-7> |
| <http://dx.doi.org/10.1016/S2213-8587(16)00044-9> |
| <http://dx.doi.org/10.1016/S2213-8587(13)70152-9> |
| <http://dx.doi.org/10.1016/S2213-8587(14)70066-X> |
| <http://dx.doi.org/10.1016/S2213-8587(13)70214-6> |
| <http://dx.doi.org/10.1016/S2213-8587(14)70251-7> |
| <http://dx.doi.org/10.1016/S2213-8587(15)00141-2> |
| <http://dx.doi.org/10.1016/S2213-8587(14)70169-X> |
| <http://dx.doi.org/10.1016/S2213-8587(13)70149-9> |
| <http://dx.doi.org/10.1016/S2213-8587(14)70200-1> |
| <http://dx.doi.org/10.1016/S2213-8587(13)70090-1> |
| 10.7326/M15-1380 |
| 10.7326/M14-2358 |
| 10.7326/M13-3005 |
| 10.7326/M14-1650 |
| 10.7326/M14-0180 |
| 10.7326/M15-1635 |
| 10.7326/M14-0611 |
| 10.7326/M14-1409 |
| 10.1001/jama.2016.11136 |
| 10.1001/jama.2014.3321 |
| 10.1001/jama.2016.1252 |
| 10.1001/jama.2014.16658 |
| 10.1001/jama.2015.9676 |
| <http://dx.doi.org/10.1016/S0140-6736(14)62115-2> |
| <http://dx.doi.org/10.1016/S0140-6736(15)60158-1> |
| <http://dx.doi.org/10.1016/S0140-6736(14)61374-X> |
| <http://dx.doi.org/10.1016/S0140-6736(14)61037-0> |
| <https://doi.org/10.1016/S0140-6736(15)60936-9> |
| <http://dx.doi.org/10.1016/S0140-6736(14)60976-4> |
| <http://dx.doi.org/10.1016/S0140-6736(14)61399-4> |
| 10.1056/NEJMoa1316222 |
| 10.1056/NEJMoa1411892 |
| 10.1056/NEJMoa1602494 |
| 10.1056/NEJMoa1504720 |
| 10.1056/NEJMoa1509351 |
| 10.1056/NEJMoa1406470 |
| 10.1056/NEJMoa1305224 |
| 10.1038/ijo.2016.52 |
| 10.1038/ijo.2015.162 |
| 10.1038/ijo.2013.224 |
| 10.1089/thy.2015.0422 |
| 10.1089/thy.2016.0010 |
| 10.1089/thy.2015.0133 |
